# The histone code reader SPIN1 controls RET signaling in liposarcoma

**DOI:** 10.18632/oncotarget.3000

**Published:** 2015-03-05

**Authors:** Henriette Franz, Holger Greschik, Dominica Willmann, Luka Ozretić, Cordula Annette Jilg, Eva Wardelmann, Manfred Jung, Reinhard Buettner, Roland Schüle

**Affiliations:** ^1^ Urologische Klinik und Zentrale Klinische Forschung, Klinikum der Universität Freiburg, Freiburg, Germany; ^2^ Universitätsklinikum Köln, Institut für Pathologie, Köln, Germany; ^3^ Universitätsklinikum Münster, Gerhard-Domagk-Insitut für Pathologie, Münster, Germany; ^4^ Institut für Pharmazeutische Wissenschaften, Albert-Ludwigs-Universität Freiburg, Freiburg, Germany; ^5^ BIOSS Centre of Biological Signaling Studies, Albert-Ludwigs-University, Freiburg, Germany; ^6^ Deutsches Konsortium für Translationale Krebsforschung (DKTK), Standort Freiburg, Germany

**Keywords:** SPIN1, histone code reader, GDNF, RET signaling, MAZ

## Abstract

The histone code reader Spindlin1 (SPIN1) has been implicated in tumorigenesis and tumor growth, but the underlying molecular mechanisms remain poorly understood. Here, we show that reducing SPIN1 levels strongly impairs proliferation and increases apoptosis of liposarcoma cells *in vitro* and in xenograft mouse models. Combining signaling pathway, genome-wide chromatin binding, and transcriptome analyses, we found that SPIN1 directly enhances expression of GDNF, an activator of the RET signaling pathway, in cooperation with the transcription factor MAZ. Accordingly, knockdown of SPIN1 or MAZ results in reduced levels of GDNF and activated RET explaining diminished liposarcoma cell proliferation and survival. In line with these observations, levels of SPIN1, GDNF, activated RET, and MAZ are increased in human liposarcoma compared to normal adipose tissue or lipoma. Importantly, a mutation of SPIN1 within the reader domain interfering with chromatin binding reduces liposarcoma cell proliferation and survival. Together, our data describe a molecular mechanism for SPIN1 function in liposarcoma and suggest that targeting SPIN1 chromatin association with small molecule inhibitors may represent a novel therapeutic strategy.

## INTRODUCTION

*SPIN1* was initially described as an abundant maternal transcript deposited in the unfertilized mouse egg [1]. The protein belongs to the SPIN/SSTY family implicated in cell cycle regulation during gametogenesis and the transition between gamete and embryo [2, 3]. Furthermore, SPIN1 was reported to be highly expressed in several types of tumors [4], and ectopic expression in cell lines was observed to affect cell cycle, chromatin segregation, or to induce apoptosis, cellular transformation, or tumor formation in nude mice [5–8]. To date, only few transcriptional targets of SPIN1 including rDNA genes and WNT/β-catenin target genes were reported [6, 9, 10] and genome-wide chromatin binding of SPIN1 has not been investigated. Thus, the precise role of SPIN1 in transcriptional control remains unclear.

SPIN1 is a histone code reader composed of three tudor-like domains [11] shown to bind histone H3 trimethylated at lysine 4 (H3K4me3) [9, 10, 12, 13], a chromatin mark typically located at promoters and associated with active or poised genes [14]. H3K4me3 peptides interact with high affinity with an aromatic pocket in the second tudor-like domain of SPIN1 [9, 13]. This association was recently shown to be further enhanced by the presence of asymmetrically dimethylated arginine 8 (H3R8me2a) [9], a mark implicated in the triggering of organizer gene expression [15]. Of note, peptides harboring only the H3R8me2a modification bind to the first tudor-like domain of SPIN1 with low affinity [9], and mutation of either F141 or Y170 in the second tudor-like domain disrupts binding of H3K4me3 as well as H3K4me3-H3R8me2a peptides [9, 10].

Liposarcoma is one of the most common types of soft tissue sarcoma and can be classified into four major histological subtypes: well-differentiated liposarcoma (WDLS), dedifferentiated liposarcoma (DDLS), myxoid liposarcoma (MLS), and pleomorphic liposarcoma (PLS) [16, 17]. Liposarcoma subtypes vary in metastatic potential and response to therapy [17]. While liposarcoma is typically treated by surgical dissection of the tumor followed by radiotherapy, there are currently no therapeutic options for aggressive and metastatic tumors [17]. Thus, there is need for new molecular therapies for treatment of aggressive liposarcoma.

One factor that has been implicated in liposarcoma is the protooncogene rearranged during transfection (RET) [18, 19]. RET is a receptor tyrosine kinase essential for normal development, differentiation, and maintenance of different cell types and tissues [20–22]. RET is activated by members of the family of glial cell-derived neurotrophic factors, which include glial cell-derived neurotrophic factor (GDNF), artemin (ARTN), neurturin (NRTN), and persephin (PSPN) [21, 22]. Glial cell-derived neurotrophic factors bind to members of the GDNF receptor alpha family (GFRA1–4) to form binary complexes. These binary complexes associate with RET inducing its dimerization and autophosphorylation. Phosphorylated RET (RETph) recruits effector proteins, which mainly activate the RAS-MAPK or the PI3K-AKT signaling pathways to control cell proliferation, differentiation, and survival [21, 22].

In this study we aimed to clarify the role of H3K4me3 binding of SPIN1 on a genome-wide scale and evaluate whether targeting SPIN1 chromatin association is a potential therapeutic strategy in cancer. We show that SPIN1 is overexpressed in human liposarcoma compared to normal adipose tissue or lipoma. Our mechanistic studies *in vitro* and in xenograft mouse models demonstrate that SPIN1, in cooperation with the transcription factor MAZ, controls proliferation and apoptosis of liposarcoma cells by directly regulating expression of the RET signaling pathway effector GDNF. Importantly, SPIN1-mediated control of target gene transcription, liposarcoma cell proliferation and survival critically depends on binding to H3K4me3 suggesting that targeting this interaction with small molecule inhibitors may be a useful therapeutic approach for cancer treatment.

## RESULTS

### SPIN1 is overexpressed in liposarcoma compared to normal adipose tissue or lipoma

**Figure 1:**
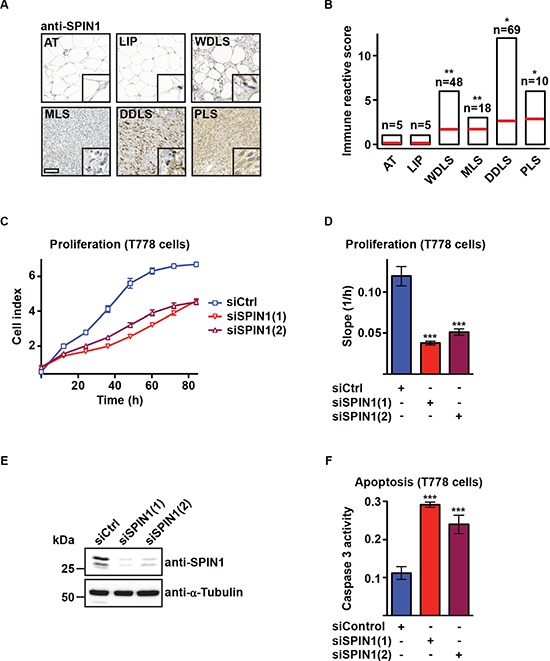
SPIN1 knockdown decreases proliferation and increases apoptosis of liposarcoma cells **(A)** Detection of SPIN1 by immunohistochemistry in adipose tissue (AT), lipoma (LIP), and liposarcoma tissue of patients [well-differentiated liposarcoma (WDLS), myxoid liposarcoma (MLS), dedifferentiated liposarcoma (DDLS), pleomorphic liposarcoma (PLS)]. Representative pictures are shown. Scale bar = 100 μm; inlay: 10x zoom. **(B)** Quantification of SPIN1 staining observed in (A) by determination of immune reactive scores for indicated numbers of patient samples. **(C, D)** Proliferation of T778 cells transfected with two different siRNAs against SPIN1 [siSPIN1(1) or siSPIN1(2)] or control siRNA (siCtrl). Growth curves (C) and slopes of exponential growth phases (D) are shown. **(E)** Western blot analysis of SPIN1 expression in T778 cells treated with the indicated siRNAs. α-Tubulin was used as loading control. **(F)** Caspase 3 activity in T778 cells transfected with siCtrl, siSPIN1(1), or siSPIN1(2). (B, D, F) Error bars represent +/– SEM, *p < 0.05, **p < 0.01, ***p < 0.001.

Screening tumor tissue arrays by immunohistochemistry with a SPIN1-specific antibody, we observed elevated SPIN1 protein levels in WDLS, MLS, DDLS, and PLS compared to normal adipose tissue or lipoma (Figure 1A, Supplementary 1A–1C). Quantification of 155 patient samples by immune reactive score showed that SPIN1 protein levels correlate with the aggressiveness of liposarcoma (Figure 1B). Furthermore, our analysis of publically available microarray data of liposaroma samples [23] confirmed that *SPIN1* mRNA significantly increases with the degree of malignancy of liposarcoma (Supplementary Figure 1D). In addition, we found strongly increased SPIN1 protein levels in the MLS-derived cell line MLS1765 [24] and the DDLS-derived cell line T778 [25, 26] compared to undifferentiated 3T3-L1 preadipocytes or differentiated adipocytes (Supplementary Figure 1E).

### Knockdown of SPIN1 decreases proliferation and increases apoptosis of liposarcoma cells

To investigate a potential role of SPIN1 in liposarcoma, we first analyzed proliferation and apoptosis of T778 and MLS1765 cells upon SPIN1 knockdown. Real-time recording of proliferating T778 cells transfected with two different siRNAs against SPIN1 [siSPIN1(1) and siSPIN1(2)] or control siRNA (siCtrl) revealed that cells proliferate more slowly upon SPIN1 depletion (Figure 1C, 1D). The efficiency of SPIN1 knockdown was verified by Western blot (Figure 1E). Furthermore, we noted significantly increased activity of caspase 3, an early marker for cell apoptosis, in T778 cells transfected with SPIN1 siRNA (Figure 1F). Increased apoptosis was verified by TUNEL assay (Supplementary Figure 1F). Comparable results were obtained upon knockdown of SPIN1 in MLS1765, SW872 [27], and T449 cells [25] (Supplementary Figure 1G–1S). Thus, SPIN1 depletion in liposarcoma cell lines results in reduced proliferation and increased apoptosis.

### The impact of SPIN1 on liposarcoma cell proliferation and apoptosis correlates with binding to H3K4me3

**Figure 2:**
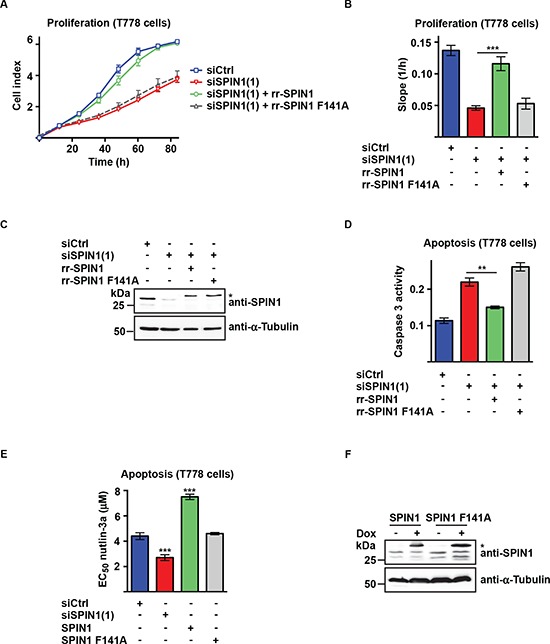
Binding of SPIN1 to H3K4me3 is required for proliferation and survival of liposarcoma cells **(A, B)** Proliferation of T778 cells transfected with control siRNA (siCtrl) or siRNA directed against SPIN1 [siSPIN1(1)] and expression plasmid for RNAi-resistant, wildtype or mutant SPIN1 (rr-SPIN1 or rr-SPIN1 F141A, respectively). Growth curves (A) and slopes of exponential growth phases (B) are shown. **(C, F)** Western blot analysis of SPIN1 expression in T778 cells transfected with siRNA and SPIN1 expression plasmid as indicated. An asterisk marks exogenous SPIN1 having a higher molecular weight than endogenous SPIN1 due to the presence of a tag. α-Tubulin was used as a loading control. In (F), SPIN1 and SPIN1 F141A expression in stably transfected T778 cells was induced by doxycycline (Dox). **(D)** Caspase 3 activity in T778 cells transfected with siRNA and SPIN1 expression plasmid as indicated. **(E)** Determination of EC_50_ values for nutlin-3a-induced apoptosis of T778 cells. Cells were transfected with siRNA and SPIN1 expression plasmid as indicated. Expression of exogenous SPIN1 was induced by doxycycline. EC_50_ values were calculated from treatment of cells with different concentrations of nutlin-3a. (B, D, E) Error bars represent +/– SEM, **p < 0.01, ***p < 0.001.

To assess if the methyl mark reader function of SPIN1 is required for the control of liposaroma cell proliferation and apoptosis, we generated a SPIN1 mutant (SPIN1 F141A) defective in binding to H3K4me3 chromatin marks [9, 10]. In addition, we generated RNAi-resistant wildtype or mutant SPIN1 (rr-SPIN1 or rr-SPIN1 F141A) to address the question, whether the effect of SPIN1 depletion on cell proliferation or survival could be rescued. Cotransfection of SPIN1 siRNA and rr-SPIN1 expression plasmid reestablished normal growth of T778 cells (Figure 2A–2C). In contrast, no rescue was observed with rr-SPIN1 F141A. Furthermore, rr-SPIN1 expression diminished caspase 3 activity in SPIN1-depleted T778 cells, whereas rr-SPIN1 F141A expression had no effect (Figure 2D). Comparable results were obtained in MLS1765 cells (Supplementary Figure 2A–2C).

To verify these results by an independent experimental approach, we transfected T778 cells with siSPIN1 or doxycycline-inducible SPIN1 or SPIN1 F141A expression plasmid and induced apoptosis by administering nutlin-3a. In T778 cells, which are characterized by amplification of the *MDM2* locus and expression of wild-type p53, nutlin-3a-induced apoptosis is p53-dependent [26, 28]. Nutlin-3a titration revealed an EC_50_ of 4.4 μM in T778 control cells (Figure 2E, 2F). In comparison, SPIN1-depleted T778 cells showed a lower EC_50_ value of 2.7 μM. Induction of SPIN1 expression increased the EC_50_ of nutlin-3a-induced apoptosis almost two-fold to 7.5 μM. In contrast, induction of SPIN1 F141A expression resulted in an EC_50_ of 4.6 μM, which is comparable to that of control cells. Thus, SPIN1 depletion sensitizes T778 cells to apoptosis whereas expression of exogenous SPIN1, but not SPIN1 F141A, protects T778 cells against nutlin-3a-induced apoptosis. Comparable results were obtained upon induction of apoptosis in MLS1765 cells, in which apoptosis cannot be induced by nutlin-3a [26], using doxorubicin (Supplementary Figure 2D, E). Together, these results show that the H3K4me3 reader function of SPIN1 is required to promote cell proliferation and reduce apoptosis of liposarcoma cells.

### SPIN1 modulates RET signaling by directly regulating *GDNF* expression

**Figure 3:**
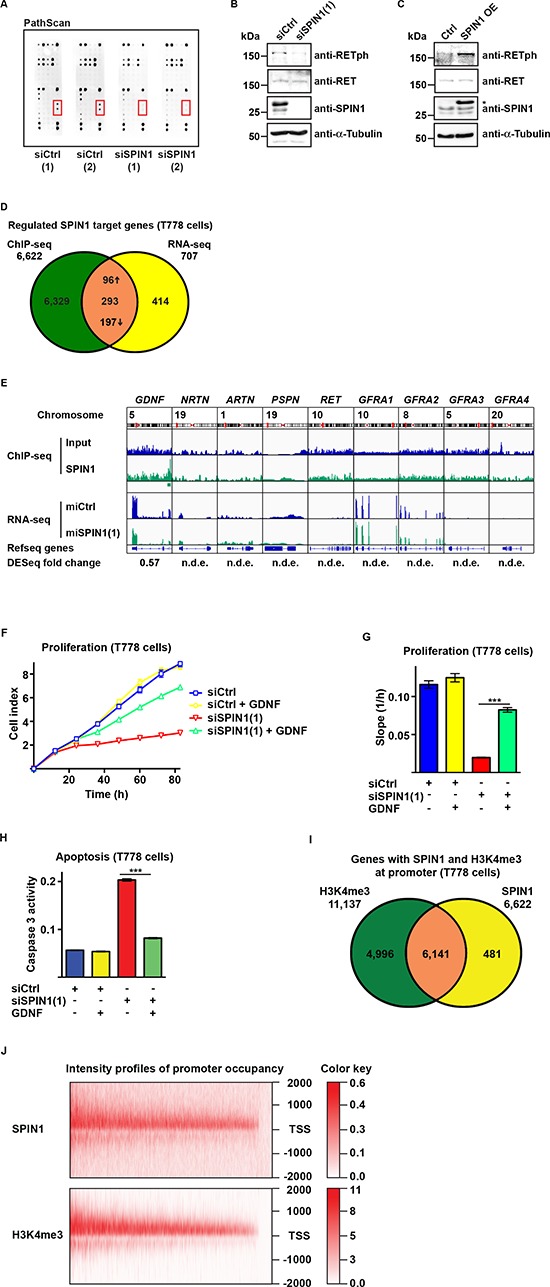
SPIN1 modulates RET signaling by controlling *GDNF* expression **(A)** PathScan analysis using extracts of T778 cells transfected with control siRNAs [siCtrl(1) or siCtrl(2)] or siRNAs directed against SPIN1 [siSPIN1(1) or siSPIN1(2)]. **(B, C)** Western blot analysis of SPIN1, RET, and phospho-RET (RETph) levels in T778 cells treated with siCtrl or siSPIN1(1) or stably transfected with SPIN1 expression plasmid (SPIN1 OE). An asterisk marks exogenous SPIN1 having a higher molecular weight than endogenous SPIN1 due to the presence of a tag. Expression of SPIN1 was induced by addition of doxycycline. α-Tubulin was used as loading control. **(D)** Venn diagram depicting the overlap of genes with SPIN1 promoter occupancy determined by ChIP-sequencing and differentially expressed genes in T778 cell upon SPIN1 depletion determined by RNA-sequencing. Up- or downregulation of genes is indicated by arrows. **(E)** Intensity profiles of presence of SPIN1 at genes involved in RET signaling in T778 cells determined by ChIP-sequencing (top) and reads determined by RNA-sequencing analysis of T778 cells stably expressing control miRNA (miCtrl) or miRNA directed against SPIN1 [miSPIN1(1)] (bottom). For *GDNF* the fold change in expression upon SPIN1 knockdown determined by DESeq is indicated. Other genes of the RET signaling pathway were not differentially expressed (n.d.e.). **(F, G)** Proliferation of T778 cells transfected with siCtrl or siSPIN1(1) in the presence or absence of GDNF. Growth curves (F) and slopes of exponential growth phases (G) are shown. **(H)** Caspase 3 activity in T778 cells treated with siCtrl or siSPIN1(1) in the presence or absence of GDNF. **(I)** Venn diagram depicting the overlap of SPIN1 and H3K4me3 locations at gene promoters in T778 cells. **(J)** Intensity profiles for SPIN1 and H3K4me3 occupancy of 6,141 gene promoters around the transcription start site (TSS –/+ 2000 bp). (G, H) Error bars represent +/– SEM, ***p < 0.001.

To identify signaling pathways involved in SPIN1-mediated control of liposarcoma cell proliferation and apoptosis, we analyzed the phosphorylation status of major signaling proteins upon siRNA-mediated knockdown of SPIN1 using a PathScan*®* RTK Signaling antibody array. PathScan analysis using T778 cell extract revealed reduced phosphorylation levels of the receptor tyrosine kinase RET upon SPIN1 knockdown (Figure 3A). Reduced levels of phosphorylated RET (RETph) in SPIN1-depleted cells were verified by Western blot (Figure 3B). Conversely, overexpression of SPIN1 increased RETph (Figure 3C). Similarly, in MLS1765 cells knockdown of SPIN1 resulted in a decrease, whereas overexpression of SPIN1 lead to an increase in RETph levels (Supplementary Figure 3A, 3B). Thus, SPIN1 levels correlate with the phosporylation status and thereby activity of RET.

The RET signaling pathway is activated by binding of the neurotrophic factors GDNF, NRTN, ARTN, or PSPN to a GFRA coreceptor (GFRA1 to 4) and subsequent complex formation with RET [21, 22]. To address the question, whether these genes are directly regulated by SPIN1, we next investigated genome-wide chromatin association of SPIN1 in T778 cells by chromatin immunoprecipitation followed by massive parallel sequencing (ChIP-seq). Our ChIP-seq analysis revealed 7,581 high-confidence SPIN1 peaks, of which 6,823 (90.0%) were located at the promoter (defined as +/− 2000 bp around the transcription start site) of 6,622 genes (Figure 3D, Supplementary Figure 3C). For the genes involved in RET signaling, we only observed a SPIN1 peak at the *GDNF* promoter (Figure 3E).

Next, we performed transcriptome analysis by massive parallel sequencing (RNA-seq) using T778 cells stably transfected with plasmid driving doxycycline-inducible expression of control miRNA or miRNA against SPIN1. Two independent miRNAs directed against SPIN1 were validated by Western blot and in proliferation assays (Supplementary Figure 3D–3H). SPIN1 depletion by miRNA(1) induced changes (≥1.5 fold, *p* ≤ 10^−15^) in the expression of 707 genes (Figure 3D, Supplementary Table 1). Intersection of the ChIP-seq and RNA-seq data revealed that 293 of the differentially expressed genes have SPIN1 promoter occupancy (Figure 3D, Supplementary Table 1). Out of these genes, 96 were up- and 197 downregulated (Figure 3D). Among the direct SPIN1 targets, *GDNF* was downregulated in SPIN1-depleted T778 cells, whereas there was no significant change in the expression of the other components of the RET signaling pathway (Figure 3E).

To verify our data from the global analyses, we first analyzed by ChIP-quantitative PCR, whether knockdown or overexpression affected SPIN1 binding to the *GDNF* promoter. In addition to primers specific for the SPIN1 binding site within the promoter region, primers in intron 3 of the *GDNF* gene not bound by SPIN1 were included as control. Indeed, siRNA-mediated knockdown specifically reduced, whereas overexpression increased SPIN1 occupancy of the *GDNF* promoter (Supplementary Figure 3I, 3J). Next, we treated T778 cells with control siRNA or siRNA directed against SPIN1 and analyzed gene expression by quantitative RT-PCR. SPIN1 knockdown resulted in downregulation of *GDNF* mRNA, whereas the mRNA level of *RET* remained unchanged (Supplementary Figure 3K). Conversely, overexpression of SPIN1 increased *GDNF*, but not *RET* mRNA (Supplementary Figure 3L). Importantly, *GDNF* expression was not affected by overexpression of SPIN1 F141A (Supplementary Figure 3M). Similar results were obtained in MLS1765 cells (Supplementary Figure 3N–3P).

To provide further evidence that compromised RET signaling is the major cause of reduced proliferation and survival of liposarcoma cells upon SPIN1 depletion, T778 cells were transfected with control siRNA or siRNA against SPIN1 and exogenous GDNF was added to the cell culture medium. GDNF supplementation antagonized reduced proliferation (Figure 3F, 3G) and increased apoptosis (Figure 3H) induced by SPIN1 knockdown to almost control levels. Similar results were obtained in MLS1765 cells (Supplementary Figure 3Q–3S). Taken together, these data demonstrate that SPIN1 directly and positively regulates *GDNF* expression and thereby controls RET signaling.

Next, we expanded our genome-wide analyses by addressing the question, whether SPIN1 promoter occupancy correlated with the presence of H3K4me3. ChIP-seq revealed 12,866 high-confidence H3K4me3 peaks, of which 11,412 (88.7%) were located at the promoter of 11,137 genes (Figure 3I, Supplementary Figure 3T). Colocalization of SPIN1 and H3K4me3 as well as overlapping intensity profiles were observed at 6,141 promoters, which corresponds to the vast majority (92.7%) of all promoters occupied by SPIN1 (Figure 3I, 3J).

### SPIN1 controls liposarcoma cell proliferation and apoptosis by modulation of RET signaling in cooperation with the transcription factor MAZ

To identify transcription factors that mediate the effect of SPIN1 on liposarcoma cell proliferation and apoptosis, we searched for enrichment of transcription factor motifs in the set of 293 differentially expressed direct SPIN1 targets. Taking into account that SPIN1 chromatin association correlates with ‘active’ H3K4me3 marks, we concentrated on the 197 genes downregulated upon SPIN1 depletion. Binding motifs of the MYC-associated zinc finger protein (MAZ) were enriched with the most significant *p*-value in the set of 197 downregulated genes, but not in the set of 96 upregulated direct SPIN1 targets (Supplementary Figure 4A). These observations indicated potential cooperation between SPIN1 and MAZ in promoting gene transcription.

**Figure 4:**
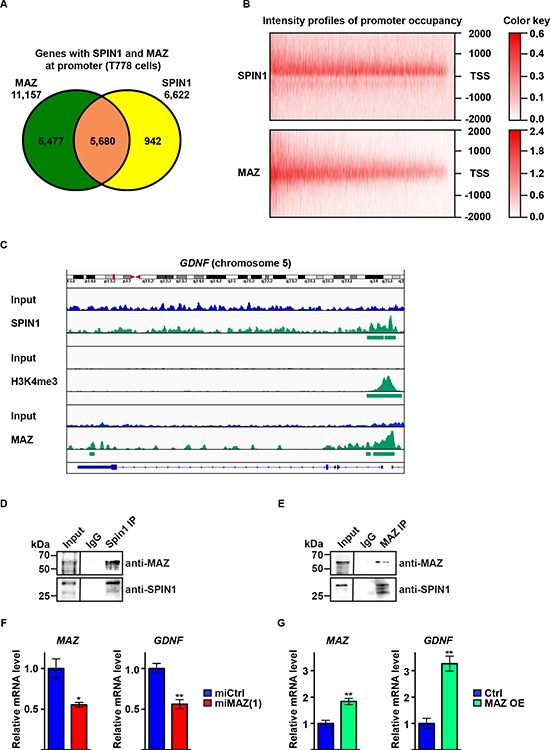
SPIN1 controls liposarcoma cell proliferation and survival by enhancing *GDNF* expression in cooperation with the transcription factor MAZ **(A)** Venn diagram depicting the overlap of SPIN1 and MAZ locations at gene promoters in T778 cells. **(B)** Intensity profiles for SPIN1 and MAZ occupancy of 5,680 gene promoters around the transcription start site (TSS –/+ 2000 bp). **(C)** Intensity profiles of presence of SPIN1, H3K4me3, and MAZ at the *GDNF* gene in T778 cells determined by ChIP-sequencing. **(D, E)** Immunoprecipitation (IP) of endogenous SPIN1 and MAZ from T778 cell extracts with antibodies against SPIN1 or MAZ as indicated. **(F, G)** Quantitative RT-PCR analysis of *MAZ* and *GDNF* expression in T778 cells stably transfected with control miRNA (miCtrl) or miRNA directed against MAZ [miMAZ(1)] (F) or MAZ expression plasmid (MAZ OE) (G) Expression of miMAZ or MAZ was induced by doxycycline. Uninduced cells served as control. (F, G) Error bars represent +/– SEM, *p < 0.05, **p < 0.01.

MAZ was initially described as a protein binding to a 16 bp region of the *MYC* promoter and has been implicated in transcriptional activation of target genes [29–31] and regulation of tumor cell proliferation and apoptosis [32, 33]. To investigate potential cooperation of SPIN1 and MAZ in liposarcoma, we analyzed genome-wide chromatin association of MAZ by ChIP-seq. Our analysis revealed 31,965 high-confidence peaks, of which 40.7% were located at the promoter of 11,157 genes (Figure 4A, Supplementary Figure 4B). Analyzing promoter occupancy by SPIN1 and MAZ, we found that MAZ was present at 5,680 out of 6,622 (85.8%) promoters occupied by SPIN1 (Figure 4A). Furthermore, the intensity profiles of promoter occupancy for both proteins overlapped well (Figure 4B). Importantly, SPIN1 and MAZ were found to colocalize with H3K4me3 at the *GDNF* promoter (Figure 4C). Specific enrichment of MAZ at the *GDNF* promoter in comparison to the control region in intron 3 of the *GDNF* gene was confirmed by ChIP-quantitative PCR (Supplementary Figure 4C).

To test whether SPIN1 and MAZ interact, we performed coimmunoprecipitation experiments using T778 cell extracts. SPIN1 antibody coprecipitated endogenous MAZ protein and MAZ antibody coprecipitated endogenous SPIN1 protein demonstrating interaction of both proteins (Figure 4D, 4E). Furthermore, Re-ChIP experiments confirmed the presence of both proteins in one complex at the *GDNF* promoter (Supplementary Figure 4D). Next, we asked whether MAZ controls *GDNF* expression. Downregulation of MAZ by two different miRNAs [miMAZ(1) or miMAZ(2)] caused a decrease in *GDNF* mRNA, while overexpression of MAZ led to an increase in *GDNF* mRNA (Figure 4F, 4G, Supplementary Figure 4E–4G). Accordingly, proliferation of T778 cells was decreased by MAZ knockdown and increased upon MAZ overexpression (Supplementary Figure 4H–4K). Finally, MAZ depletion lowered the EC_50_ for nutlin-3a-induced apoptosis and increased caspase 3 activity in T778 cells (Supplementary Figure 4L, 4M). In comparison, MAZ overexpression reduced the sensitivity of T778 to nutlin-3a-induced apoptosis (Supplementary Figure 4L). Together, these data suggest that MAZ and SPIN1 cooperate to regulate *GDNF* expression in T778 cells and that MAZ, at least in part, contributes to SPIN1-mediated control of liposarcoma cell proliferation and apoptosis.

### SPIN1 controls tumor proliferation and apoptosis in mice

**Figure 5:**
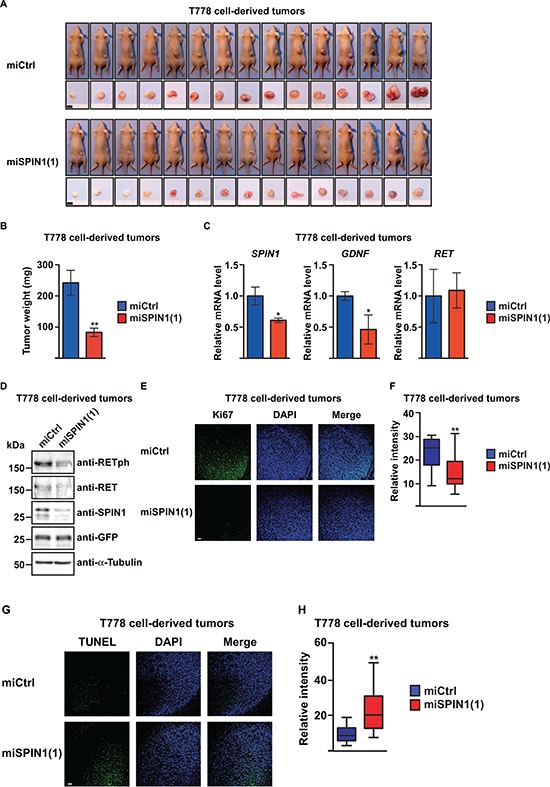
SPIN1 controls proliferation and apoptosis of liposarcoma cell-derived tumors in BALB/c nude mice **(A)** Analysis of tumors from BALB/c nude mice (*n* = 15) 10 days after subcutaneous injection of T778 cells expressing control miRNA (miCtrl) or miRNA against SPIN1 [miSPIN1(1)]. Scale bar = 5 mm. **(B)** Average tumor weight of mice shown in (A) **(C)** Quantitative RT-PCR analysis of *SPIN1*, *GDNF*, and *RET* expression in T778 cell-derived tumors treated with the indicated miRNA. **(D)** Western blot analysis of SPIN1, RET, and RETph levels in T778 cell-derived tumors treated with the indicated miRNA. α-Tubulin and GFP were used as loading controls. **(E)** Detection of Ki67 by immunofluorescence in T778 cell-derived tumors treated with the indicated miRNA. Scale bar = 100 μm. **(F)** Quantification of Ki67 staining shown in (E). **(G)** TUNEL assay for detection of apoptotic cells in T778 cell-derived tumors treated with the indicated miRNA. Scale bar = 100 μm. **(H)** Quantification of TUNEL staining shown in (G) (B, C, F, H) Error bars represent +/– SEM, *p < 0.05, **p < 0.01.

T778 cells have previously been shown to induce tumors when injected into nude mice [34]. To test the effect of SPIN1 depletion on tumorigenic activity *in vivo*, we performed xenograft assays with T778 cells stably transfected with plasmid driving doxycycline-inducible expression of control miRNA or miRNA directed against SPIN1 concomitant with expression of GFP. The efficiency of SPIN1 knockdown was controlled by quantitative RT-PCR and Western blot (Supplementary Figure 5A, 5B). Cells were injected subcutaneously into BALB/c nude mice after 48 hours of doxycycline treatment, and subsequently the mice orally received doxycycline every second day. After ten days, tumors were analyzed (Figure 5A).

The weight of the tumors derived from SPIN1 knockdown cells was significantly reduced compared to control tumors (Figure 5B). Staining with SPIN1 antibody confirmed reduced SPIN1 levels in tumors derived from T778 cells treated with SPIN1 miRNA (Supplementary Figure 5C, 5D). We next analyzed expression of *SPIN1*, *GDNF*, and *RET* in the tumors by quantitative RT-PCR. In SPIN1-depleted tumors, mRNA levels of *SPIN1* and *GDNF* were significantly reduced, whereas expression of *RET* remained unchanged (Figure 5C), which is consistent with observations in cell culture (Supplementary Figure 5A). Western blot analysis confirmed unchanged levels of RET, but revealed lower levels of RETph in SPIN1-depleted compared to control tumors (Figure 5D). Comparable results were obtained in a xenograft mouse model using MLS1765 cells (Supplementary Figure 5E–5H). Thus, SPIN1 knockdown reduces liposarcoma cell-derived tumor growth in mice, which correlates with reduced levels of both *GDNF* mRNA and RET phosphorylation.

Next, we aimed to understand whether the reduced tumor size observed in BALB/c nude mice resulted from decreased proliferation and/or increased apoptosis of SPIN1-depleted liposarcoma cells. Staining with antibody against Ki67, a marker for proliferating cells, revealed that in SPIN1-depleted T778 cell-derived tumors the number of proliferating cells was strongly reduced (Figure 5E, 5F). Similar observations were made for tumors derived from stably transfected MLS1765 cells (Supplementary Figure 5I, 5J). Furthermore, TUNEL assays revealed that the number of apoptotic cells within tumors derived from T778 or MLS1765 cells was higher in SPIN1-depleted compared to control tumors (Figure 5G, 5H, Supplementary Figure 5K, 5L). Hence, SPIN1 depletion reduces proliferation and increases apoptosis of tumors derived from liposarcoma cells *in vivo*.

### Levels of GDNF, phosphorylated RET, and MAZ are increased in liposarcoma samples of patients

**Figure 6:**
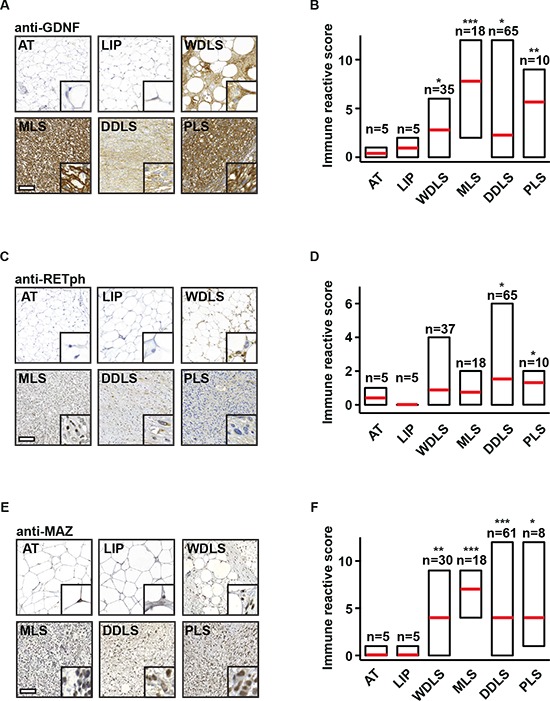
Levels of GDNF, phosphorylated RET, and MAZ are increased in liposarcoma samples of patients **(A)** Detection of GDNF by immunohistochemistry in normal adipose tissue (AT), lipoma (LIP), and liposarcoma tissues [well-differentiated liposarcoma (WDLS), myxoid liposarcoma (MLS), dedifferentiated liposarcoma (DDLS), pleomorphic liposarcoma (PLS)]. Representative pictures are shown. Scale bar = 100 μm, inlay: 10× zoom. **(B)** Quantification of GDNF staining observed in (A) by determination of immune reactive scores for indicated numbers of patient samples. **(C)** Detection of phospho-RET (RETph) by immunohistochemistry in normal AT, LIP, and liposarcoma tissues (WDLS, MLS, DDLS, PLS). Representative pictures are shown. Scale bar = 100 μm, inlay: 10× zoom. **(D)** Quantification of RETph staining observed in (C) by determination of immune reactive scores for indicated numbers of patient samples. **(E)** Detection of MAZ by immunohistochemistry in normal AT, LIP, and liposarcoma tissues. Representative pictures are shown. Scale bar = 100 μm, inlay: 10× zoom. **(F)** Quantification of MAZ staining observed in (E) by determination of immune reactive scores for indicated numbers of patient samples. (B, D, F) Error bars represent +/− SEM, *p < 0.05, **p < 0.01, ***p < 0.001.

To investigate the relevance of our observations for human disease, we stained tissue samples from liposarcoma patients with GDNF, RETph, or MAZ antibody and determined the immune reactive scores. Analysis of 155 patient samples revealed that levels of GDNF, activated RET (RETph), and MAZ were elevated in all four types of liposarcoma (Figure 6A–6F). Furthermore, staining of adipose tissue, lipoma, WDLS, MLS, DDLS, or PLS samples taken from one patient with SPIN1, GNDF, RETph, or MAZ antibody showed that also in individual patients high levels of SPIN1 correlate with high levels of GDNF, RETph, or MAZ (Supplementary Figure 6).

In summary, our data demonstrate that SPIN1 controls proliferation and survival of liposarcoma by regulating *GDNF* expression and thereby RET activation.

## DISCUSSION

In this study, we identified SPIN1 as a potential therapeutic target. SPIN1 is overexpressed in human liposarcoma compared to normal adipose tissue or lipoma and enhances proliferation and restricts apoptosis of tumor cells. Our mechanistic studies show that SPIN1 controls proliferation and apoptosis by activating the RET signaling pathway through direct regulation of *GDNF* expression, which depends on the binding of SPIN1 to H3K4me3. Furthermore, SPIN1 cooperates with the transcription factor MAZ in the control of *GDNF* expression. In accordance with these results, we observe increased levels of GDNF, activated RET, and MAZ in human liposarcoma compared to normal adipose tissue or lipoma.

Tudor-like domain 2 of SPIN1 binds with high affinity to H3K4me3 [9, 10, 13], a chromatin mark typically observed at active or poised promoters [14]. H3K4me3 binding was recently shown to be enhanced by association of tudor-like domain 1 with H3R8me2a [9]. However, compared to high affinity H3K4me3 binding, SPIN1 associates with peptides carrying only the H3R8me2a mark with dramatically reduced affinity [9]. Furthermore, SPIN1 binding to peptides carrying H3K4me3 and H3R8me2 marks is strongly impaired by a F141A mutation in the aromatic cage of tudor-like domain 2 [9]. Thus, H3K4me3 binding seems to be the major determinant of SPIN1 transcriptional functions at chromatin. This idea is in line with our experiments demonstrating that SPIN1, but not SPIN1 F141A, can rescue the effect of SPIN1 depletion on liposarcoma cell proliferation and apoptosis.

Our data provide evidence that SPIN1 controls liposarcoma cell proliferation and apoptosis by directly enhancing the expression of GDNF, an activator of the RET signaling pathway. This idea is corroborated by our genome-wide study of SPIN1 chromatin association combined with the analysis of transcriptome changes upon SPIN1 depletion. These analyses show that among the components of the RET signaling pathway, *GDNF* is the only direct transcriptional target of SPIN1. Thus, SPIN1 controls target gene expression, proliferation, and apoptosis by modulating RET signaling in liposarcoma.

In our genome-wide binding analysis, we detected SPIN1 at the promoter of 6,622 genes, at most of which SPIN1 colocalized with H3K4me3. Despite the presence of SPIN1 and H3K4me3 at a vast number of gene promoters, only the minority of occupied genes is differentially up- or downregulated upon SPIN1 knockdown. These observations suggest that SPIN1-mediated transcriptional control does not only require binding to H3K4me3, but is determined by additional factors. Searching for transcription factors targets in the set of genes differentially expressed upon SPIN1 knockdown, we identified MAZ, a transcription factor initially observed to bind to a 16 bp region in the *MYC* promoter and implicated in transcriptional activation [29–31]. Furthermore, MAZ is upregulated in prostate tumors and positively regulates androgen receptor transcription [32], and MAZ depletion was reported to lead to reduced proliferation and increased apoptosis of prostate or breast cancer cells [32, 33]. MAZ is present at the majority of promoters occupied by SPIN1. Both proteins interact and regulate *GDNF* gene expression in cooperation. Thus, in addition to previously reported TCF4 [6, 9], our data identify the transcription factor MAZ acting in concert with SPIN1 in the control of target gene transcription.

High levels of SPIN1 have been observed in liposarcoma (this study) and other types of tumors including ovarian cancer [4]. To our knowledge, to date, mechanisms leading to increased expression of SPIN1 in tumors have not been elucidated. In future studies, it will be interesting to investigate and compare mechanisms such as gene amplification, increased de novo mRNA synthesis, or mRNA stability, which may cause SPIN1 overexpression in different types of tumors.

While liposarcoma is a relatively rare disease, which is typically treated by surgical dissection and radiotherapy, there are currently no therapeutic options for aggressive and metastatic tumors [17]. Since RET has been implicated in liposarcoma [18, 19], it is considered a potential therapeutic target. However, efforts to target RET specifically with small molecule inhibitors such as Vandetanib or Cabozantinib in e.g. thyroid or lung adenocarcinoma have only shown limited success [22, 35]. This is in part due to moderate target specificity, since Vandetanib also inhibits VEGFR2 and EGFR [36], and Cabozantinib also targets MET and VEGFR2 [37].

Recently, histone code readers have emerged as a novel class of potential drug targets. Inhibitors of reader domains are prominently exemplified by JQ1 or I-BET, compounds potently disrupting the interaction of BET bromo domains with acetylated lysine residues [38, 39]. Furthermore, successful targeting of a methyl lysine reader (L3MBTL3A) with the small molecule inhibitor UNC1215 has been reported [40] and several other methyl lysine readers are potential drug targets [41]. Since the F141A mutation in tudor-like domain 2, which blocks SPIN1 chromatin binding, interferes with SPIN1-controlled liposarcoma cell proliferation and survival, targeting the SPIN1/H3K4me3 pocket with small molecule inhibitors might be an interesting alternative therapeutic option for cancer treatment.

## METHODS

### Generation of SPIN1 antibody

For anti-SPIN1(1) antibody generation Glutathione-S-transferase- (GST-) tagged SPIN1 protein (amino acids 183–229) was expressed in E.coli BL21 and affinity purified using Glutathione Sepharose 4B resin (GE Healthcare Life Sciences) in buffer containing 20 mM Tris pH 8.0, 200 mM NaCl. Protein was eluted from affinity resin in purification buffer supplemented with 20 mM glutathione. Purity of GST-SPIN1(183–229) protein was estimated to be greater than 95% by SDS-PAGE. For a second antibody [anti-SPIN1(2)] hexahistidine- (His-) tagged SPIN1 protein (amino acids 49–262) was expressed in E.coli BL21 and affinity-purified using TALON resin (Clontech) in buffer containing 20 mM Tris pH 8.0, 200 mM NaCl. Protein was eluted from TALON resin using purification buffer supplemented with 50 mM imidazole pH 8.0. Purity of His-SPIN1(49–262) protein was estimated to be greater than 95% by SDS-PAGE. Antibodies were generated by injection of purified protein into rabbits (Biogenes, Berlin) using standard technology. For Western blot and ChIP anti-SPIN1(1) was applied. For immunohistochemistry and immunofluorescence anti-SPIN1(2) was used.

### Plasmids

The following plasmids were used: pCMX_SPIN1_Flag_HA, pCMX_SPIN1_F141A_Flag_HA, pSLIC_Neo_Flag_HA_SPIN1, pSLIC_Neo_Flag_HA_SPIN1_F141A, pRTS_Puro_miRNA_Control, pRTS_Puro_miRNA_ SPIN1(1), pRTS_Puro_miRNA_SPIN1(2), pRTS_Puro_miRNA_MAZ(1), pRTS_Puro_miRNA_MAZ(2), pRTS_Puro_MAZ. The pSLIC_Neo plasmids allow doxycycline-inducible expression of SPIN1 or SPIN1 F141A. The pRTS_Puro plasmids contain a doxycycline-inducible bidirectional promoter driving expression of miRNA or protein and GFP. For expression in E.coli the following plasmids were used: pET15b-SPIN1(49–262) and pGST-SPIN1(183–229), a modified pET15b derivative in which the His cassette was replaced with a GST cassette. Cloning strategies and details of plasmids will be provided upon request.

### Cell culture

T778, MLS1765, SW872, and T449 cells were cultured in RPMI medium supplemented with 10% FCS, penicillin/streptomycin and glutamine. Cells were transfected in 6-well-plates with siRNA using RNAiMax applying standard protocols (Life Technologies), with siRNA and pCMX_SPIN1_Flag_HA or pCMX_SPIN1_F141A_Flag_HA expression plasmid using Dharmafect Duo (Thermo Scientific), or with pRTS_Puro_miRNA vector using Fugene HD (Promega). Sequences of siRNAs and miRNAs are provided in Supplementary Table 2. For selection, cells stably transfected with pRTS_Puro_miRNA vector were cultured in medium containing 1 μg/ml puromycin. Expression of miRNA and GFP was induced with 0.5 μg/ml doxycycline. Cell lines allowing inducible expession of wildtype or mutant SPIN1 were established by lentiviral infection as described [42] applying 10 μg/ml polybrene and subsequent selection with 500 μg/ml neomycin. SPIN1 expression was induced with 0.5 μg/ml doxycycline.

### Chromatin immunoprecipitation and ChIP-sequencing

Chromatin immunoprecipitation (ChIP) was performed as described [42]. Briefly, cells were crosslinked with 1% formaldehyde for 20 min at 4°C. Cells were washed with PBS, harvested in lysis buffer [50 mM Tris pH 8.0, 1 mM EDTA, 1% SDS, 1x protease inhibitor cocktail (11873580001, Roche)], and sonified with Bioruptor (Diagenode) to 200–500 bp fragmented DNA. Per ChIP experiment 5 μg of rabbit anti-SPIN1(1), anti-H3K4me3 (ab8580, Abcam), or anti-MAZ (NB100–86984, Novus Biological) antibody were used. After washing, immunoprecipitated DNA was eluted with buffer containing 0.1 M sodium hydrogen carbonate and 1% SDS for 1 h at room temperature. The crosslinks were reversed by incubation at 65°C overnight. Finally, the DNA was purified using the MinElute PCR purification Kit (Qiagen). Sequence reads from each ChIP-seq library were sequenced on an Illumina platform (Illumina). 49 bp sequences were generated and mapped to the hg19 genome by bowtie [43]. These raw sequencing data were further analyzed using the peak finding algorithm MACS [44] using sequences from input as control. All peaks with a false discovery rate less than 2% were included. The uniquely mapping locations were used to generate the genome-wide intensity profiles, which were visualized using the IGV genome browser [45]. HOMER [46] was used to annotate peaks and calculate overlaps between different bed files. The ChIP-sequencing data from this publication have been submitted to the GEO database (http://www.ncbi.nlm.nih.gov/geo/) and assigned the identifier GSE57502.

### RNA-sequencing and quantitative RT-PCR

RNA was isolated with Trizol (Life Technologies) and treated with DNAse (M6101, Promega) according to the company's instructions. For RNA-sequencing (RNA-seq), RNA quality was determined by RNA 6000 Nano Kit technology (5067–15119, Agilent). RNA with a RIN above 8 was sequenced at the DKFZ core facility (Heidelberg, Germany) using Illumina technology [47]. For the RNA-seq cleaned sequenced paired-end reads were mapped to the human reference genome (hg19) using TopHat software (http://tophat.cbcb.umd.edu/) [48]. To identify the differentially expressed genes, the reads for RefSeq annotated transcripts were counted with HOMER software [46] and differentially expressed genes were calculated with the DESeq package [49]. Differentially expressed genes with *p* ≤ 10^−15^ and a fold change ≥1.5 were used for further analysis. The RNA-seq data from this publication have been submitted to the GEO database (http://www.ncbi.nlm.nih.gov/geo/) and assigned the identifier GSE57502. The genes obtained from the ChIP-seq and RNA-seq analysis were further analysed using a WebGestalt KEGG analysis or a WebGestalt transcription factor motif search [50, 51]. For quantitative RT-PCR cDNA was prepared by reverse transcription of mRNA using Superscript II (Life Technologies). Quantitative RT-PCR was performed with Lightcycler 480 II (Roche) using Absolute SYBR green ROX Mix (Thermo Scientific). Primers used are listed in Supplementary Table 2.

### Western blot and PathScan analysis

SDS PAGE and Western blot were performed according to standard protocols. The following antibodies were used: anti-SPIN1(1) (1:1000), anti-alpha-Tubulin (1:20000, T6074, Sigma), anti-RET (1:100, ab134100, Abcam), anti-RETph (1:200, ab51103, Abcam), anti-GFP (1:5000, ab6556, Abcam), anti-MAZ (1:100, H-50, Santa Cruz). The Pathscan analysis was conducted using the PathScan RTK Signaling Antibody Array Kit (7949S, Cell Signaling). Arrays were probes according to the manufacturers instructions with 150 μl (0.5 μg/μl) of extract of T778 cells transfected with control siRNA or siRNA directed against SPIN1.

### Immunoprecipitation

T778 cells were harvested in extraction buffer (50 mM Tris pH 8.0, 170 mM NaCl, 0.1% NP40, 20% glycerol, 50 mM NaF, 2 mM NaVanadate) and were sonified 3 × 30s with Sonorex RK52 (Bandelin). For immunoprecipitation 5 μg of anti-Spin1(1) or anti-MAZ (H-50, Santa Cruz) antibody were crosslinked to GammaBind sepharose (17–0885-01, GE Healthcare) according to the Abcam protocol (http://www.abcam.com/ps/pdf/protocols/crosslinking.pdf). 1 mg of protein extract was incubated with beads for 2 h. Afterwards beads were washed 3 × 5 min with extraction buffer and eluted with glycine (100 mM, pH 2.5) for 15 min at room temperature. Eluates were used for Western blot.

### Proliferation assay

Proliferation of T778 and MLS1765 cells was determined using the X-Celligence RTCA system (Roche). For real-time recording of T778, MLS1765, or T449 cell proliferation, 2500 cells/well were seeded in 16-well E-plates (Roche). For SW872 5000 cells/well were used. For rescue experiments, cells were treated with 50 ng /ml GDNF for 24 h prior to seeding in E-plates. This concentration was kept during the assays.

### Immunofluorescence and immunohistochemistry

For immunofluorescence staining, paraffin sections of tumors were deparaffinized and for antigen retrieval heated in 20 mM Tris (pH 9.0) for 20 min in a pressure cooker. Sections were blocked for 20 min at room temperature in 5% FCS/PBST (0.1% Triton X-100). SPIN1(2) antibody (1:100) or Ki67 antibody (1:20, NB110–89717, Novus Biologicals) were applied at 4°C overnight. After washing, sections were incubated with secondary antibody [goat anti-rabbit Alexa 488, 1:5000 in PBST (0.1% Triton X-100), A11034, Molecular Probes] for 1 h at room temperature. Sections were washed four times for 5 min with PBST (0.1% Triton X-100) and nuclei were stained with DAPI (1 μg/ml) for 6 min at room temperature followed by two washing steps with PBS. Then sections were mounted with Fluoromount (Sigma). Tissue microarrays were prepared from 155 formalin-fixed, paraffin-embedded liposarcoma or control samples. All tumors had been staged by two independent experienced pathologists. Two different tissue cores from single tumors were arrayed from formalin-fixed, paraffin-embedded tissue blocks using a manual device (Beecher Instruments). Four-micrometer paraffin sections were cut from every tissue microarray and used for immunohistochemical staining. Immunohistochemical staining was performed using the following antibodies: anti-SPIN1(2) (1:100), anti-RETph (1:100, ab51103, Abcam), anti-GDNF (1:50, ab18956, Abcam), and anti-MAZ (1:150, ab83397, Abcam). The results were evaluated by a semiquantitative scoring system as described [52].

### Apoptosis assays

For TUNEL assay of cells or paraffin sections the In Situ Cell Death Detection Kit (11684795910, Roche) was used. Cells were fixed with 4% PFA in PBS (pH 7.4), washed twice with PBS, permeabilized for two minutes with 0.1% Triton X-100 in 0.1% sodium citrate, and washed again twice with PBS. DNase treated cells and sections were used as positive control. Nuclei were stained with DAPI (0.5 μg/ml). Pictures were obtained using a Leica SP2 confocal microscope (Leica Microsystems). The signal intensity of the TUNEL assay was quantified using ImageJ [53] and normalized to the DAPI signal. Caspase 3 activity was determined using the Caspase3/CPP32 Colorimetric Protease Assay Kit (Life Technologies). Briefly, cells were harvested, lysed, and cell debris was pelleted by centrifugation at 2000 rpm. 50 μg of the supernatant were used for the assay. Absorbance was measured at 405 nm. For rescue experiments, cells were treated with 50 ng/ml GDNF for 24 h. This concentration was kept during the assays.

### Xenograft assay

T778 or MLS1765 cells were stably transfected with inducible miRNA expression plasmid (pRTS_Puro_miRNA_Control or pRTS_Puro_miRNA_SPIN1). Expression of miRNA and GFP (from a bidirectional promoter) was induced with 0.5 μg/ml doxycycline two days before subcutaneous injection. 2 × 10^6^ T778 cells per mouse were injected subcutaneously into BALB/c nude mice. For continued miRNA expression, mice received a dose of 10 μg doxycycline per g of body weight every second day for ten days. Then, mice were sacrificed and tumors isolated. Tumor weight was determined using a fine balance (BP121S, Satorius). For MLS1765 cells, which are known to give rise to tumors only occasionally, different cell numbers (from 1 × 10^5^ up to 1 × 10^7^ per mouse) were injected subcutaneously. Initial tumors were isolated from mice, minced, and incubated with isolation buffer (25 mg DNase, 100 mg hyaluronidase, 400 mg collagenase in HBSS buffer) for 15 min at 37°C. Supernatant with separated cells was centrifuged for 5 min at 800 rpm. This step was repeated three times, cell pellets were pooled, and cells cultured in RPMI medium containing 10% FCS supplemented with penicillin/streptomycin and glutamine. MLS1765 tumor cells obtained by this procedure were stably transfected with inducible miRNA expression plasmid (pRTS_Puro_miRNA_Control or pRTS_Puro_miRNA_SPIN1) and 2 × 10^5^ cells per mouse were used for Xenograft assay. Mice received 10 μg doxycycline per g of body weight every second day for 30 days.

## SUPPLEMENTARY FIGURES AND TABLES





## Abbreviations

(AT): adipose tissue
(LIP): lipoma
(WDLS): well-differentiated liposarcoma
(MLS): myxoid liposarcoma
(DDLS): dedifferentiated liposarcoma
(PLS): pleomorphic liposarcoma
(KD): knockdown
(OE): overexpression
(rr): RNAi-resistant
(Dox): doxycycline
