# Supplementary material for: The histone code reader SPIN1 controls RET signaling in liposarcoma
Source: Oncotarget. 2015 Mar 5;6(7):4773–89. doi: 10.18632/oncotarget.3000 (PMC4467114; doi:10.18632/oncotarget.3000)
Supplement: Supplementary file 1 [file oncotarget-06-4773-s001.pdf]

Enfocus
PitStop
Server

Preflight Report

Errors were found on page 1-15 and in general document properties

- PDF/X output intent is missing
- PDF/X version is not PDF/X-1a:2001
- Resolution of color or grayscale image is less than 300 dpi (106x on pages 1-5,9,11-13)
- Font Arial-BoldMT, TimesNewRomanPSMT, Arial-BoldItalicMT is a composite font (4123x on pages 1-15)
- Line weight is less than 0.5 pt (2x on pages 1,11)
- Line weight is less than 0.5 pt (2x on pages 1,11)
- Embedded font TimesNewRomanPS-ItalicMT, TimesNewRomanPS-BoldMT, Symbol-Bold, TimesNewRomanPSMT, Arial-BoldMT, Symbol, TimesNewRomanPS-BoldItalicMT, Arial-BoldItalicMT is not complete (4318x on pages 1-15)

Warnings

- Indexed color is used (75x on pages 1-5,7-12)
- Font Verdana-Bold, TimesNewRomanPSMT, TimesNewRomanPS-ItalicMT, TimesNewRomanPS-BoldMT, TimesNewRomanPS-BoldItalicMT is a TrueType font (211x on pages 1-15)
- Resolution of color or grayscale image is more than 450 dpi (1x on page 10)
- Total ink coverage of flat color is more than 310% (11x on page 13)

Enfocus PitStop Server

General File Information

Processing

Preflight Profile	diacriTech McVety-kendel Settings
Created by	Hari Raj<br>diacriTech Pvt. Ltd.
Pages processed	1-15
Application used to preflight	Enfocus PitStop Server 09, update 3 powered by Enfocus PitStop Library 09, update 3
Application used to create the report	Enfocus PitStop Library 09, update 3

Document properties

Document Name	Supplementary.pdf
Number of pages	15
PDF Version	1.4
Creation Date	UTC: February 5, 2015 - 07:14:14 [February 5, 2015 - 12:44:14]
Modification Date	UTC: February 5, 2015 - 07:14:36 [February 5, 2015 - 12:44:36]
Producer	Adobe PDF Library 9.9
Creator	Adobe InDesign CS5.5 (7.5.3)
Author	Top
Title	Microsoft Word - 100710.docx
Subject	-
Keywords	-
Trapped	no
Trapping Information Present	no
Repaired on Open	no
Optimized for Fast Web View	yes
Thumbnails Present	no
Binding	left
Job Ticket Present	no

Security

Encryption Type	not encrypted
Content Copying or Extraction	allowed
Content Access for Disabled Users	allowed
Adding or Modifying Annotations	allowed
Creating or Modifying Form Fields	allowed
Form Field Fill-in or Signing	allowed
Document Assembly	allowed
Other Document Modifications	allowed
Printing	high resolution

Compression

Data format	binary
Document structure compressed	yes
Filters used	JPEG, ZIP

Page Boxes

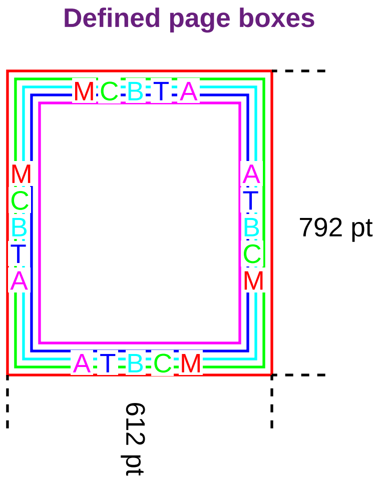

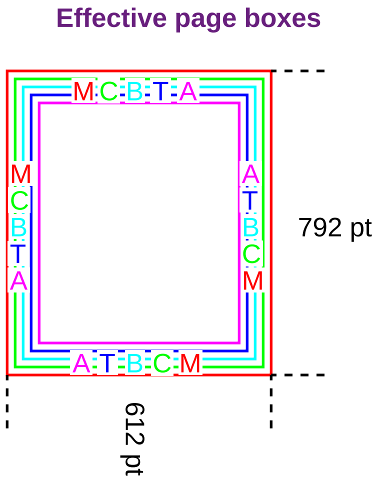

Page box	Defined size	Effective size
Media box (M)	612 x 792 pt	612 x 792 pt
Crop box (C)	612 x 792 pt	612 x 792 pt
Trim box (T)	612 x 792 pt	612 x 792 pt
Bleed box (B)	612 x 792 pt	612 x 792 pt
Art box (A)	612 x 792 pt	612 x 792 pt

Defined for pages 1-15
Media box starts at : (0, 0) pt
Page rotation : 0 degrees

Enfocus PitStop Server

Font Information

PostScript font name	Type	Embedded as	Subset	Encoding
Verdana-Bold	TrueType	TrueType	no	WinAnsi
TimesNewRomanPS-BoldMT	TrueType	TrueType	yes	WinAnsi
Arial-BoldMT	Composite TT	TrueType	yes	Identity-H
Symbol-Bold	Type 1	Type 1	yes	Custom
TimesNewRomanPSMT	TrueType	TrueType	yes	WinAnsi
TimesNewRomanPSMT	Composite TT	TrueType	yes	Identity-H
TimesNewRomanPS-ItalicMT	TrueType	TrueType	yes	WinAnsi
Symbol	Type 1	Type 1	yes	Custom
TimesNewRomanPS-BoldItalicMT	TrueType	TrueType	yes	WinAnsi
Arial-BoldItalicMT	Composite TT	TrueType	yes	Identity-H

Enfocus PitStop Server

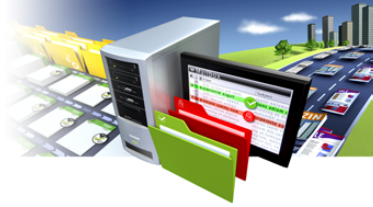

Image Information

Type	Color Space	Size (pixels)	Resolution (dpi)	Bits per channel	Page	Angle (degrees)	Skew	Flipped	Custom Transfer	Custom Halftone	Custom BG	Custom UCR
Color	CMYK	495x322	300.3x300.5	8	1	0	-	-	-	-	-	-
Color	Indexed	105x424	228.6x278.4	8	1	0	-	-	-	-	-	-
Color	Indexed	106x380	230.7x249.5	8	1	0	-	-	-	-	-	-
Color	CMYK	184x184	301.2x301.2	8	1	0	-	-	-	-	-	-
Color	CMYK	184x184	301.2x301.2	8	1	0	-	-	-	-	-	-
Color	CMYK	184x184	301.2x301.2	8	1	0	-	-	-	-	-	-
Color	CMYK	184x184	301.2x301.2	8	1	0	-	-	-	-	-	-
Color	CMYK	184x184	301.2x301.2	8	1	0	-	-	-	-	-	-
Color	CMYK	184x184	301.2x301.2	8	1	0	-	-	-	-	-	-
Color	CMYK	183x184	300.3x301.2	8	1	0	-	-	-	-	-	-
Color	CMYK	183x184	300.3x301.2	8	1	0	-	-	-	-	-	-
Color	CMYK	183x183	300.3x300.3	8	1	0	-	-	-	-	-	-
Color	CMYK	184x183	301.2x300.3	8	1	0	-	-	-	-	-	-
Color	Indexed	205x205	335.6x336.4	8	1	0	-	-	-	-	-	-
Color	Indexed	287x86	209.3x355.2	8	1	0	-	-	-	-	-	-
Color	Indexed	297x64	216.3x267.8	8	1	0	-	-	-	-	-	-
Color	Indexed	139x61	202.2x248.8	8	1	0	-	-	-	-	-	-
Color	Indexed	141x54	205.6x220.3	8	1	0	-	-	-	-	-	-
Color	CMYK	244x321	300.2x300	8	1	0	-	-	-	-	-	-
Color	CMYK	331x349	300.3x300	8	1	0	-	-	-	-	-	-
Color	CMYK	184x184	301.2x301.2	8	1	0	-	-	-	-	-	-
Color	CMYK	184x184	301.2x301.2	8	1	0	-	-	-	-	-	-
Color	CMYK	184x184	301.2x301.2	8	1	0	-	-	-	-	-	-
Color	CMYK	184x184	301.2x301.2	8	1	0	-	-	-	-	-	-
Color	CMYK	184x184	301.2x301.2	8	1	0	-	-	-	-	-	-
Color	CMYK	184x184	301.2x301.2	8	1	0	-	-	-	-	-	-
Color	CMYK	184x184	301.2x301.2	8	1	0	-	-	-	-	-	-
Color	CMYK	185x185	301.6x301.6	8	2	0	-	-	-	-	-	-
Color	CMYK	185x185	301.6x301.6	8	2	0	-	-	-	-	-	-
Color	CMYK	185x185	301.6x301.6	8	2	0	-	-	-	-	-	-
Color	CMYK	185x185	301.6x301.6	8	2	0	-	-	-	-	-	-
Color	CMYK	333x351	300.9x300.5	8	2	0	-	-	-	-	-	-
Color	CMYK	245x323	300.1x300.6	8	2	0	-	-	-	-	-	-
Color	Indexed	141x54	204.7x219.4	8	2	0	-	-	-	-	-	-
Color	Indexed	139x61	201.4x247.8	8	2	0	-	-	-	-	-	-
Color	Indexed	297x64	215.4x266.6	8	2	0	-	-	-	-	-	-
Color	Indexed	287x86	208.4x353.8	8	2	0	-	-	-	-	-	-
Color	Indexed	205x205	334.2x335	8	2	0	-	-	-	-	-	-
Color	CMYK	185x184	301.6x300.7	8	2	0	-	-	-	-	-	-
Color	CMYK	184x185	300.7x301.6	8	2	0	-	-	-	-	-	-
Color	CMYK	184x185	300.7x301.6	8	2	0	-	-	-	-	-	-

Image Information

Type	Color Space	Size (pixels)	Resolution (dpi)	Bits per channel	Page	Angle (degrees)	Skew	Flipped	Custom Transfer	Custom Halftone	Custom BG	Custom UCR
Color	CMYK	185x185	301.6x301.6	8	2	0	-	-	-	-	-	-
Color	CMYK	185x185	301.6x301.6	8	2	0	-	-	-	-	-	-
Color	Indexed	106x380	229.8x248.5	8	2	0	-	-	-	-	-	-
Color	Indexed	105x424	227.6x277.3	8	2	0	-	-	-	-	-	-
Color	CMYK	497x323	300.2x300.2	8	2	0	-	-	-	-	-	-
Color	CMYK	185x185	300.8x300.8	8	2	0	-	-	-	-	-	-
Color	CMYK	184x185	300x300.8	8	2	0	-	-	-	-	-	-
Color	CMYK	184x185	300x300.8	8	2	0	-	-	-	-	-	-
Color	CMYK	185x184	300.8x300	8	2	0	-	-	-	-	-	-
Color	CMYK	185x184	300.8x300	8	2	0	-	-	-	-	-	-
Color	CMYK	184x184	300x300	8	2	0	-	-	-	-	-	-
Color	CMYK	184x184	300x300	8	2	0	-	-	-	-	-	-
Color	CMYK	184x184	300x300	8	2	0	-	-	-	-	-	-
Color	CMYK	184x184	300x300	8	2	0	-	-	-	-	-	-
Color	CMYK	277x372	300.1x300.5	8	2	0	-	-	-	-	-	-
Color	CMYK	277x372	300.1x300.5	8	2	0	-	-	-	-	-	-
Color	CMYK	480x343	300.3x300.8	8	2	0	-	-	-	-	-	-
Color	Indexed	122x343	301.6x300.8	8	2	0	-	-	-	-	-	-
Color	Indexed	407x8	300.5x312.5	8	2	0	-	-	-	-	-	-
Color	Indexed	82x26	300.2x307.6	8	2	0	-	-	-	-	-	-
Color	Indexed	83x32	300.2x300.7	8	2	0	-	-	-	-	-	-
Color	Indexed	83x32	300.2x305.5	8	2	0	-	-	-	-	-	-
Color	Indexed	171x67	247.5x272.1	8	2	0	-	-	-	-	-	-
Color	Indexed	172x59	249x239.6	8	2	0	-	-	-	-	-	-
Color	CMYK	493x343	300.2x300.8	8	2	0	-	-	-	-	-	-
Color	CMYK	124x343	300.3x300.8	8	2	0	-	-	-	-	-	-
Color	CMYK	250x401	301x300.2	8	2	0	-	-	-	-	-	-
Color	CMYK	246x323	301.1x300.1	8	2	0	-	-	-	-	-	-
Color	CMYK	243x320	300.5x300.4	8	3	0	-	-	-	-	-	-
Color	CMYK	275x368	301x300.3	8	3	0	-	-	-	-	-	-
Color	CMYK	275x368	301x300.3	8	3	0	-	-	-	-	-	-
Color	CMYK	475x339	300.3x300.4	8	3	0	-	-	-	-	-	-
Color	Indexed	121x339	302.2x300.4	8	3	0	-	-	-	-	-	-
Color	Indexed	403x8	300.7x315.8	8	3	0	-	-	-	-	-	-
Color	Indexed	82x26	303.3x310.8	8	3	0	-	-	-	-	-	-
Color	Indexed	83x32	303.3x303.8	8	3	0	-	-	-	-	-	-
Color	Indexed	83x32	303.3x308.7	8	3	0	-	-	-	-	-	-
Color	Indexed	171x67	250.1x275	8	3	0	-	-	-	-	-	-
Color	Indexed	172x59	251.6x242.1	8	3	0	-	-	-	-	-	-
Color	CMYK	488x339	300.2x300.4	8	3	0	-	-	-	-	-	-
Color	CMYK	123x339	301x300.4	8	3	0	-	-	-	-	-	-

Enfocus PitStop Server

Image Information

Type	Color Space	Size (pixels)	Resolution (dpi)	Bits per channel	Page	Angle (degrees)	Skew	Flipped	Custom Transfer	Custom Halftone	Custom BG	Custom UCR
Color	CMYK	247x397	300.5x300.3	8	3	0	-	-	-	-	-	-
Color	CMYK	183x183	300.7x301.5	8	3	0	-	-	-	-	-	-
Color	CMYK	183x183	300.7x301.5	8	3	0	-	-	-	-	-	-
Color	CMYK	183x183	301.5x300.7	8	3	0	-	-	-	-	-	-
Color	CMYK	183x183	301.5x300.7	8	3	0	-	-	-	-	-	-
Color	CMYK	272x364	301x300.7	8	3	0	-	-	-	-	-	-
Color	Indexed	171x76	232.5x290.1	8	3	0	-	-	-	-	-	-
Color	Indexed	182x67	247.5x255.7	8	3	0	-	-	-	-	-	-
Color	CMYK	669x389	300.2x300.3	8	4	0	-	-	-	-	-	-
Color	CMYK	400x422	300.3x300.3	8	4	0	-	-	-	-	-	-
Color	CMYK	389x389	300.3x300.3	8	4	0	-	-	-	-	-	-
Color	CMYK	400x422	300.3x300.3	8	4	0	-	-	-	-	-	-
Color	Indexed	197x88	178x296.3	8	4	0	-	-	-	-	-	-
Color	Indexed	198x63	178.9x213.3	8	4	0	-	-	-	-	-	-
Color	Indexed	94x47	186.6x174.9	8	5	0	-	-	-	-	-	-
Color	Indexed	93x55	186.5x207.3	8	5	0	-	-	-	-	-	-
Color	Indexed	109x53	216.4x201	8	5	0	-	-	-	-	-	-
Color	Indexed	109x53	218.6x196.1	8	5	0	-	-	-	-	-	-
Color	Indexed	101x71	200.5x264.3	8	5	0	-	-	-	-	-	-
Color	Indexed	92x51	182.7x188.7	8	5	0	-	-	-	-	-	-
Color	Indexed	111x53	220.4x202.3	8	5	0	-	-	-	-	-	-
Color	Indexed	98x48	194.6x177.6	8	5	0	-	-	-	-	-	-
Color	CMYK	354x353	300.8x300.3	8	5	0	-	-	-	-	-	-
Color	Indexed	199x46	197.9x170.2	8	5	0	-	-	-	-	-	-
Color	Indexed	200x58	198.9x214.5	8	5	0	-	-	-	-	-	-
Color	CMYK	554x354	300.5x300.8	8	5	0	-	-	-	-	-	-
Color	CMYK	554x353	300.5x300.3	8	5	0	-	-	-	-	-	-
Color	CMYK	186x354	301.5x300.8	8	5	0	-	-	-	-	-	-
Color	CMYK	186x353	301.5x300.3	8	5	0	-	-	-	-	-	-
Color	CMYK	193x365	301.4x300.7	8	6	0	-	-	-	-	-	-
Color	CMYK	193x365	301.4x300.7	8	6	0	-	-	-	-	-	-
Color	CMYK	193x365	301.4x300.7	8	6	0	-	-	-	-	-	-
Color	CMYK	193x365	301.4x300.7	8	6	0	-	-	-	-	-	-
Color	CMYK	192x365	300.6x300.7	8	6	0	-	-	-	-	-	-
Color	CMYK	192x365	300.6x300.7	8	6	0	-	-	-	-	-	-
Color	CMYK	193x365	301.4x300.7	8	6	0	-	-	-	-	-	-
Color	CMYK	193x365	301.4x300.7	8	6	0	-	-	-	-	-	-
Color	CMYK	185x358	301.5x300.3	8	6	0	-	-	-	-	-	-
Color	CMYK	185x358	301.5x300.3	8	6	0	-	-	-	-	-	-
Color	CMYK	185x358	300.7x300.3	8	6	0	-	-	-	-	-	-
Color	CMYK	186x358	301.5x300.3	8	6	0	-	-	-	-	-	-

Image Information

Enfocus PitStop Server

	Type	Color Space	Size (pixels)	Resolution (dpi)	Bits per channel	Page	Angle (degrees)	Skew	Flipped	Custom Transfer	Custom Halftone	Custom BG	Custom UCR
	Color	CMYK	185x358	300.7x300.3	8	6	0	-	-	-	-	-	-
	Color	CMYK	185x358	300.7x300.3	8	6	0	-	-	-	-	-	-
	Color	CMYK	187x360	301.5x300.3	8	7	0	-	-	-	-	-	-
	Color	CMYK	187x360	301.5x300.3	8	7	0	-	-	-	-	-	-
	Color	CMYK	187x360	301.5x300.3	8	7	0	-	-	-	-	-	-
	Color	CMYK	194x391	301.4x300.3	8	7	0	-	-	-	-	-	-
	Color	CMYK	194x391	301.4x300.3	8	7	0	-	-	-	-	-	-
	Color	Indexed	163x7	301.7x318.2	8	7	0	-	-	-	-	-	-
	Color	CMYK	194x391	301.4x300.3	8	7	0	-	-	-	-	-	-
	Color	Indexed	103x11	302.6x311.3	8	7	0	-	-	-	-	-	-
	Color	CMYK	187x361	301.5x300.7	8	7	0	-	-	-	-	-	-
	Color	CMYK	187x361	301.5x300.7	8	7	0	-	-	-	-	-	-
	Color	CMYK	187x360	301.5x300.3	8	7	0	-	-	-	-	-	-
	Color	CMYK	194x391	301.4x300.3	8	7	0	-	-	-	-	-	-
	Color	CMYK	194x391	301.4x300.3	8	7	0	-	-	-	-	-	-
	Color	CMYK	194x391	301.4x300.3	8	7	0	-	-	-	-	-	-
	Color	CMYK	366x386	300.7x300.7	8	8	0	-	-	-	-	-	-
	Color	Indexed	134x6	302x321.4	8	8	0	-	-	-	-	-	-
	Color	Indexed	29x15	309.6x319.2	8	8	0	-	-	-	-	-	-
	Color	Indexed	147x28	300.8x304.3	8	8	0	-	-	-	-	-	-
	Color	Indexed	147x35	300.8x303.5	8	8	0	-	-	-	-	-	-
	Color	Indexed	90x29	301.3x309.6	8	8	0	-	-	-	-	-	-
	Color	Indexed	147x35	300.8x303.5	8	8	0	-	-	-	-	-	-
	Color	Indexed	134x392	302x300.3	8	8	0	-	-	-	-	-	-
	Color	CMYK	659x392	300.2x300.3	8	8	0	-	-	-	-	-	-
	Color	CMYK	357x356	300.8x300.8	8	8	0	-	-	-	-	-	-
	Color	CMYK	366x386	300.7x300.3	8	8	0	-	-	-	-	-	-
	Color	CMYK	200x397	301.4x300.3	8	9	0	-	-	-	-	-	-
	Color	Indexed	94x9	301.3x314	8	9	0	-	-	-	-	-	-
	Color	CMYK	205x387	301.3x300.3	8	9	0	-	-	-	-	-	-
	Color	CMYK	192x387	301.4x300.3	8	9	0	-	-	-	-	-	-
	Color	CMYK	192x387	301.4x300.3	8	9	0	-	-	-	-	-	-
	Color	CMYK	357x357	300.8x300.3	8	9	0	-	-	-	-	-	-
	Color	Indexed	153x82	300.8x301.5	8	9	0	-	-	-	-	-	-
	Color	Indexed	216x110	424.6x401.9	8	9	0	-	-	-	-	-	-
	Color	Indexed	178x80	349.9x292.3	8	9	0	-	-	-	-	-	-
	Color	Indexed	153x82	300.8x301.5	8	9	0	-	-	-	-	-	-
	Color	CMYK	182x367	301.5x300.3	8	10	0	-	-	-	-	-	-
	Color	CMYK	636x367	300.2x300.3	8	10	0	-	-	-	-	-	-
	Color	Indexed	57x27	302.1x304.5	8	10	0	-	-	-	-	-	-
	Color	Indexed	72x26	301.7x304.7	8	10	0	-	-	-	-	-	-

Enfocus PitStop Server

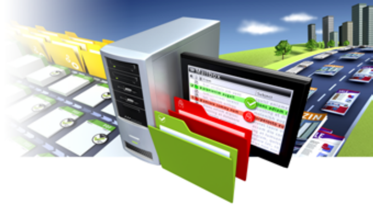

Image Information

Type	Color Space	Size (pixels)	Resolution (dpi)	Bits per channel	Page	Angle (degrees)	Skew	Flipped	Custom Transfer	Custom Halftone	Custom BG	Custom UCR
Color	Indexed	391x7	300.3x318.2	8	10	0	-	-	-	-	-	-
Color	CMYK	182x367	301.5x300.7	8	10	0	-	-	-	-	-	-
Color	CMYK	617x367	300.4x300.3	8	10	0	-	-	-	-	-	-
Color	CMYK	182x367	300.7x300.7	8	10	0	-	-	-	-	-	-
Color	Indexed	361x3	596.4x428.6	8	10	0	-	-	-	-	-	-
Color	CMYK	274x367	301x300.3	8	10	0	-	-	-	-	-	-
Color	CMYK	284x405	301x300.3	8	10	0	-	-	-	-	-	-
Color	CMYK	198x405	301.4x300.3	8	10	0	-	-	-	-	-	-
Color	Indexed	119x79	248.5x305.3	8	11	0	-	-	-	-	-	-
Color	Indexed	117x57	242.7x221.6	8	11	0	-	-	-	-	-	-
Color	Indexed	77x51	159.7x199.5	8	11	0	-	-	-	-	-	-
Color	Indexed	107x54	222.7x211.3	8	11	0	-	-	-	-	-	-
Color	Indexed	104x53	215.7x204.8	8	11	0	-	-	-	-	-	-
Color	CMYK	175x338	301.2x300.4	8	11	0	-	-	-	-	-	-
Color	CMYK	175x338	301.2x300.4	8	11	0	-	-	-	-	-	-
Color	CMYK	175x338	301.2x300.4	8	11	0	-	-	-	-	-	-
Color	CMYK	175x339	300.4x300.9	8	11	0	-	-	-	-	-	-
Color	CMYK	142x78	300.5x303.3	8	11	0	-	-	-	-	-	-
Color	CMYK	145x78	300.7x303.3	8	11	0	-	-	-	-	-	-
Color	Indexed	99x46	206.7x182.3	8	11	0	-	-	-	-	-	-
Color	Indexed	93x49	192.9x194.1	8	11	0	-	-	-	-	-	-
Color	Indexed	99x57	205.3x224.4	8	11	0	-	-	-	-	-	-
Color	CMYK	80x180	305.4x280.5	8	11	0	-	-	-	-	-	-
Color	CMYK	80x180	305.4x280.5	8	11	0	-	-	-	-	-	-
Color	CMYK	80x180	305.4x279.8	8	11	0	-	-	-	-	-	-
Color	CMYK	80x180	305.4x279.8	8	11	0	-	-	-	-	-	-
Color	CMYK	80x180	305.4x279.8	8	11	0	-	-	-	-	-	-
Color	CMYK	80x180	305.4x279.8	8	11	0	-	-	-	-	-	-
Color	CMYK	80x180	305.4x279.8	8	11	0	-	-	-	-	-	-
Color	CMYK	80x180	305.4x279.8	8	11	0	-	-	-	-	-	-
Color	CMYK	80x180	305.4x279.8	8	11	0	-	-	-	-	-	-
Color	CMYK	80x180	305.4x279.8	8	11	0	-	-	-	-	-	-
Color	CMYK	80x180	305.4x280.5	8	11	0	-	-	-	-	-	-
Color	CMYK	80x180	305.4x280.5	8	11	0	-	-	-	-	-	-
Color	CMYK	80x180	305.4x280.5	8	11	0	-	-	-	-	-	-
Color	CMYK	80x180	305.4x280.5	8	11	0	-	-	-	-	-	-
Color	CMYK	80x180	305.4x280.5	8	11	0	-	-	-	-	-	-
Color	CMYK	60x60	229x229	8	11	0	-	-	-	-	-	-
Color	CMYK	60x60	229x229	8	11	0	-	-	-	-	-	-
Color	CMYK	60x60	229x229	8	11	0	-	-	-	-	-	-
Color	CMYK	60x60	229x229	8	11	0	-	-	-	-	-	-

Enfocus PitStop Server

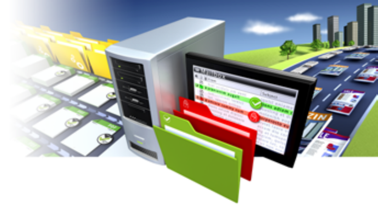

Image Information

Type	Color Space	Size (pixels)	Resolution (dpi)	Bits per channel	Page	Angle (degrees)	Skew	Flipped	Custom Transfer	Custom Halftone	Custom BG	Custom UCR
Color	CMYK	60x60	229x229	8	11	0	-	-	-	-	-	-
Color	CMYK	60x60	229x229	8	11	0	-	-	-	-	-	-
Color	CMYK	60x60	229x229	8	11	0	-	-	-	-	-	-
Color	CMYK	60x60	229x229	8	11	0	-	-	-	-	-	-
Color	CMYK	60x60	229x229	8	11	0	-	-	-	-	-	-
Color	CMYK	60x60	229x229	8	11	0	-	-	-	-	-	-
Color	CMYK	60x60	229x229	8	11	0	-	-	-	-	-	-
Color	CMYK	60x60	229x229	8	11	0	-	-	-	-	-	-
Color	CMYK	60x60	229x229	8	11	0	-	-	-	-	-	-
Color	CMYK	193x193	300x300.8	8	11	0	-	-	-	-	-	-
Color	CMYK	284x284	441.5x441.5	8	11	0	-	-	-	-	-	-
Color	CMYK	284x284	441.5x442.6	8	11	0	-	-	-	-	-	-
Color	CMYK	284x284	442.6x441.5	8	11	0	-	-	-	-	-	-
Color	CMYK	284x284	442.6x442.6	8	11	0	-	-	-	-	-	-
Color	CMYK	193x193	300x300.8	8	11	0	-	-	-	-	-	-
Color	CMYK	60x60	229x229	8	11	0	-	-	-	-	-	-
Color	CMYK	60x60	229x229	8	11	0	-	-	-	-	-	-
Color	CMYK	60x60	229x229	8	11	0	-	-	-	-	-	-
Color	CMYK	60x60	229x229	8	11	0	-	-	-	-	-	-
Color	CMYK	60x60	229x229	8	11	0	-	-	-	-	-	-
Color	CMYK	80x180	305.4x280.5	8	11	0	-	-	-	-	-	-
Color	CMYK	80x180	307.3x280.5	8	11	0	-	-	-	-	-	-
Color	CMYK	80x180	305.4x280.5	8	11	0	-	-	-	-	-	-
Color	CMYK	80x190	305.4x295.4	8	11	0	-	-	-	-	-	-
Color	CMYK	80x190	305.4x295.4	8	11	0	-	-	-	-	-	-
Color	CMYK	60x60	229x229	8	11	0	-	-	-	-	-	-
Color	CMYK	80x80	305.4x305.4	8	11	0	-	-	-	-	-	-
Color	CMYK	205x205	305.8x305.8	8	12	0	-	-	-	-	-	-
Color	Indexed	205x205	306.6x305.8	8	12	0	-	-	-	-	-	-
Color	CMYK	205x205	306.6x305.8	8	12	0	-	-	-	-	-	-
Color	CMYK	205x205	305.8x305.8	8	12	0	-	-	-	-	-	-
Color	Indexed	205x205	306.6x305.8	8	12	0	-	-	-	-	-	-
Color	Indexed	205x205	306.6x305.8	8	12	0	-	-	-	-	-	-
Color	CMYK	202x201	301.3x300.6	8	12	0	-	-	-	-	-	-
Color	CMYK	202x201	301.3x300.6	8	12	0	-	-	-	-	-	-
Color	CMYK	201x201	300.6x300.6	8	12	0	-	-	-	-	-	-
Color	CMYK	201x201	300.6x300.6	8	12	0	-	-	-	-	-	-
Color	CMYK	284x284	424.7x424.7	8	12	0	-	-	-	-	-	-
Color	CMYK	284x284	424.7x424.7	8	12	0	-	-	-	-	-	-
Color	CMYK	189x383	300.6x300.7	8	12	0	-	-	-	-	-	-
Color	CMYK	189x383	300.6x300.7	8	12	0	-	-	-	-	-	-

Enfocus PitStop Server

Image Information

Type	Color Space	Size (pixels)	Resolution (dpi)	Bits per channel	Page	Angle (degrees)	Skew	Flipped	Custom Transfer	Custom Halftone	Custom BG	Custom UCR
Color	Indexed	88x42	177.1x157.3	8	12	0	-	-	-	-	-	-
Color	Indexed	102x44	203.2x163.8	8	12	0	-	-	-	-	-	-
Color	Indexed	108x41	215.1x152.6	8	12	0	-	-	-	-	-	-
Color	Indexed	98x31	195.2x115.4	8	12	0	-	-	-	-	-	-
Color	CMYK	151x81	300.8x301.5	8	12	0	-	-	-	-	-	-
Color	CMYK	189x383	300.6x300.7	8	12	0	-	-	-	-	-	-
Color	CMYK	67x67	211.4x211.4	8	13	0	-	-	-	-	-	-
Color	CMYK	251x252	318.3x319.5	8	13	0	-	-	-	-	-	-
Color	CMYK	252x252	318.9x318.9	8	13	0	-	-	-	-	-	-
Color	CMYK	252x252	319.5x319.5	8	13	0	-	-	-	-	-	-
Color	CMYK	267x260	337.8x329.7	8	13	0	-	-	-	-	-	-
Color	CMYK	72x72	227.1x227.1	8	13	0	-	-	-	-	-	-
Color	CMYK	72x72	227.1x227.1	8	13	0	-	-	-	-	-	-
Color	CMYK	67x67	211.4x211.4	8	13	0	-	-	-	-	-	-
Color	CMYK	67x67	212.5x211.4	8	13	0	-	-	-	-	-	-
Color	CMYK	67x67	211.4x211.4	8	13	0	-	-	-	-	-	-
Color	CMYK	67x67	211.4x211.4	8	13	0	-	-	-	-	-	-
Color	CMYK	67x67	211.4x211.4	8	13	0	-	-	-	-	-	-
Color	CMYK	252x252	318.9x319.5	8	13	0	-	-	-	-	-	-
Color	CMYK	252x252	319.5x319.5	8	13	0	-	-	-	-	-	-
Color	CMYK	252x252	319.5x319.5	8	13	0	-	-	-	-	-	-
Color	CMYK	267x260	337.8x329.7	8	13	0	-	-	-	-	-	-
Color	CMYK	72x72	228.3x227.1	8	13	0	-	-	-	-	-	-
Color	CMYK	267x260	337.8x329.7	8	13	0	-	-	-	-	-	-
Color	CMYK	72x72	227.1x227.1	8	13	0	-	-	-	-	-	-
Color	CMYK	72x72	227.1x227.1	8	13	0	-	-	-	-	-	-
Color	CMYK	72x72	227.1x227.1	8	13	0	-	-	-	-	-	-
Color	CMYK	72x72	227.1x227.1	8	13	0	-	-	-	-	-	-
Color	CMYK	267x260	337.8x329.7	8	13	0	-	-	-	-	-	-
Color	CMYK	72x72	227.1x227.1	8	13	0	-	-	-	-	-	-
Color	CMYK	72x72	227.1x227.1	8	13	0	-	-	-	-	-	-
Color	CMYK	72x72	227.1x227.1	8	13	0	-	-	-	-	-	-
Color	CMYK	72x72	227.1x227.1	8	13	0	-	-	-	-	-	-
Color	CMYK	72x72	227.1x227.1	8	13	0	-	-	-	-	-	-
Color	CMYK	267x260	338.5x329.7	8	13	0	-	-	-	-	-	-
Color	CMYK	267x260	337.8x329	8	13	0	-	-	-	-	-	-
Color	CMYK	267x260	337.8x329	8	13	0	-	-	-	-	-	-
Color	CMYK	267x260	337.8x329	8	13	0	-	-	-	-	-	-
Color	CMYK	267x260	337.8x329	8	13	0	-	-	-	-	-	-
Color	CMYK	267x260	338.5x329	8	13	0	-	-	-	-	-	-
Color	CMYK	67x67	211.4x211.4	8	13	0	-	-	-	-	-	-

Image Information

Type	Color Space	Size (pixels)	Resolution (dpi)	Bits per channel	Page	Angle (degrees)	Skew	Flipped	Custom Transfer	Custom Halftone	Custom BG	Custom UCR
Color	CMYK	67x67	211.4x211.4	8	13	0	-	-	-	-	-	-
Color	CMYK	67x67	211.4x211.4	8	13	0	-	-	-	-	-	-
Color	CMYK	67x67	211.4x211.4	8	13	0	-	-	-	-	-	-
Color	CMYK	67x67	211.4x211.4	8	13	0	-	-	-	-	-	-
Color	CMYK	67x67	211.4x211.4	8	13	0	-	-	-	-	-	-
Color	CMYK	242x252	306.8x319.5	8	13	0	-	-	-	-	-	-
Color	CMYK	252x252	318.9x319.5	8	13	0	-	-	-	-	-	-
Color	CMYK	252x252	319.5x319.5	8	13	0	-	-	-	-	-	-
Color	CMYK	250x252	317x318.9	8	13	0	-	-	-	-	-	-
Color	CMYK	250x252	316.3x318.9	8	13	0	-	-	-	-	-	-
Color	CMYK	250x252	317x318.9	8	13	0	-	-	-	-	-	-
Color	CMYK	267x260	338.5x329	8	13	0	-	-	-	-	-	-
Color	CMYK	267x260	337.8x329	8	13	0	-	-	-	-	-	-

OPI Information

No OPI

Enfocus PitStop Server

Color Information

Item	Value
Color Space	Indexed
Page	1-2
Base Space	CMYK
Range	233
Color Space	Indexed
Page	1-2
Base Space	CMYK
Range	254
Color Space	Indexed
Page	1-2
Base Space	CMYK
Range	226
Color Space	Indexed
Page	1-2
Base Space	CMYK
Range	250
Color Space	Indexed
Page	1-2
Base Space	CMYK
Range	253
Color Space	CMYK
Page	1-15
Color Space	Indexed
Page	1-2
Base Space	CMYK
Range	203
Color Space	Indexed
Page	1-2
Base Space	CMYK
Range	221
Color Space	Indexed
Page	2-4
Base Space	CMYK
Range	255
Color Space	Indexed
Page	2-3,7-10

Item	Value
Base Space	CMYK
Range	0
Color Space	Indexed
Page	2
Base Space	CMYK
Range	219
Color Space	Indexed
Page	2-3
Base Space	CMYK
Range	156
Color Space	Indexed
Page	3
Base Space	CMYK
Range	253
Color Space	Indexed
Page	3
Base Space	CMYK
Range	248
Color Space	Indexed
Page	3
Base Space	CMYK
Range	226
Color Space	Indexed
Page	4
Base Space	CMYK
Range	247
Color Space	Indexed
Page	5
Base Space	CMYK
Range	121
Color Space	Indexed
Page	5
Base Space	CMYK
Range	254
Color Space	Indexed
Page	5

Enfocus PitStop Server

Color Information

Item	Value
Base Space	CMYK
Range	216
Color Space	Indexed
Page	5
Base Space	CMYK
Range	208
Color Space	Indexed
Page	5
Base Space	CMYK
Range	231
Color Space	Indexed
Page	5
Base Space	CMYK
Range	190
Color Space	Indexed
Page	5
Base Space	CMYK
Range	221
Color Space	Indexed
Page	5
Base Space	CMYK
Range	223
Color Space	Indexed
Page	5
Base Space	CMYK
Range	252
Color Space	Indexed
Page	5
Base Space	CMYK
Range	253
Color Space	Indexed
Page	7
Base Space	CMYK
Range	8
Color Space	Indexed
Page	8

Enfocus PitStop Server

Color Information

Item	Value
Base Space	CMYK
Range	0
Color Space	Indexed
Page	9
Base Space	CMYK
Range	253
Color Space	Indexed
Page	9
Base Space	CMYK
Range	87
Color Space	Indexed
Page	9
Base Space	CMYK
Range	87
Color Space	Pattern
Page	9
Pattern Type	Colored Tile
Color Space	Indexed
Page	9
Base Space	CMYK
Range	254
Color Space	Pattern
Page	9
Pattern Type	Colored Tile
Color Space	Indexed
Page	11
Base Space	CMYK
Range	242
Color Space	Indexed
Page	11
Base Space	CMYK
Range	250
Color Space	Indexed
Page	11
Base Space	CMYK
Range	181

Color Information

Item	Value
Color Space	Indexed
Page	11
Base Space	CMYK
Range	250
Color Space	Indexed
Page	11
Base Space	CMYK
Range	253
Color Space	Indexed
Page	11
Base Space	CMYK
Range	178
Color Space	Indexed
Page	11
Base Space	CMYK
Range	192
Color Space	Indexed
Page	11
Base Space	CMYK
Range	105
Color Space	Indexed
Page	12
Base Space	CMYK
Range	233
Color Space	Indexed
Page	12
Base Space	CMYK
Range	225
Color Space	Indexed
Page	12
Base Space	CMYK
Range	98
Color Space	Indexed
Page	12
Base Space	CMYK
Range	156

Enfocus PitStop Server

Color Information

Item	Value
Color Space	Indexed
Page	12
Base Space	CMYK
Range	196
Color Space	Indexed
Page	12
Base Space	CMYK
Range	250
Color Space	Indexed
Page	12
Base Space	CMYK
Range	222

Output Intents

No output intents
